# Increased CSF levels of soluble AXL at diagnosis correlate with poor prognosis in patients affected by amyotrophic lateral sclerosis

**DOI:** 10.1093/braincomms/fcag086

**Published:** 2026-03-30

**Authors:** Mauro G Spatafora, Jonas Dubin, Teuta Domi, Raffaella Lombardi, Paolo Cabras, Eleonora Dalla Bella, Monica Consonni, Angelo Quattrini, Manuela Verri, Giuseppe Lauria, Philip Van Damme, Koen Poesen, Nilo Riva, Marco Peviani

**Affiliations:** Cellular and Molecular Neuropharmacology Laboratory, Department of Biology and Biotechnology ‘L. Spallanzani’, University of Pavia, Pavia, Italy; Laboratory for Molecular Neurobiomarker Research, Department of Neurosciences, Leuven Brain Institute, KU Leuven (University of Leuven), Leuven, Belgium; Laboratory Medicine, UZ Leuven (University Hospitals Leuven), Leuven, Belgium; Experimental Neuropathology Unit, Institute of Experimental Neurology (INSPE), Division of Neuroscience, IRCCS San Raffaele Scientific Institute, Milan, Italy; 3rd Neurology Unit—Motor Neuron Disease Centre, Fondazione IRCCS Istituto Neurologico C. Besta, Milano, Italy; Cellular and Molecular Neuropharmacology Laboratory, Department of Biology and Biotechnology ‘L. Spallanzani’, University of Pavia, Pavia, Italy; 3rd Neurology Unit—Motor Neuron Disease Centre, Fondazione IRCCS Istituto Neurologico C. Besta, Milano, Italy; 3rd Neurology Unit—Motor Neuron Disease Centre, Fondazione IRCCS Istituto Neurologico C. Besta, Milano, Italy; Experimental Neuropathology Unit, Institute of Experimental Neurology (INSPE), Division of Neuroscience, IRCCS San Raffaele Scientific Institute, Milan, Italy; Cellular and Molecular Neuropharmacology Laboratory, Department of Biology and Biotechnology ‘L. Spallanzani’, University of Pavia, Pavia, Italy; 3rd Neurology Unit—Motor Neuron Disease Centre, Fondazione IRCCS Istituto Neurologico C. Besta, Milano, Italy; Department of Medical Biotechnology and Translational Medicine, University of Milan, Milan, Italy; Department of Neurology, UZ Leuven (University Hospitals Leuven), Leuven, Belgium; Laboratory of Neurobiology, Department of Neurosciences, Leuven Brain Institute, KU Leuven (University of Leuven), Leuven, Belgium; Laboratory for Molecular Neurobiomarker Research, Department of Neurosciences, Leuven Brain Institute, KU Leuven (University of Leuven), Leuven, Belgium; Laboratory Medicine, UZ Leuven (University Hospitals Leuven), Leuven, Belgium; 3rd Neurology Unit—Motor Neuron Disease Centre, Fondazione IRCCS Istituto Neurologico C. Besta, Milano, Italy; Cellular and Molecular Neuropharmacology Laboratory, Department of Biology and Biotechnology ‘L. Spallanzani’, University of Pavia, Pavia, Italy

**Keywords:** biomarker, disease progression, neurodegeneration, neuroinflammation

## Abstract

AXL, a receptor tyrosine kinase expressed in neurons and glial cells, involved in neuronal survival, myelination, and regulation of immune responses, can undergo shedding due to the activation of metalloproteases in neuroinflammatory conditions. Indeed, CSF and serum levels of soluble AXL (sAXL) have been correlated with neurodegeneration and cognitive decline in Alzheimer’s disease (AD). Based on these observations, we explored whether sAXL is implicated in amyotrophic lateral sclerosis (ALS). sAXL levels were measured in biofluids (CSF and serum) from two biorepositories, totalling 107 ALS patients, 76 healthy controls, 25 AD patients, 22 patients with multiple sclerosis and 51 patients with ALS disease mimicking disorders (i.e. patients that displayed symptoms resembling ALS, in whom eventually ALS was excluded after a thorough clinical examination). Gender and age were considered as covariate in the statistical analyses. Our results provide the first evidence of sAXL alterations in the CSF and serum of ALS patients at diagnosis and demonstrate a significant association between CSF sAXL levels and disease progression, as well as its prognostic value in ALS. While these observations require validation through multicentre studies, they suggest the involvement of the AXL pathway in ALS pathology and pave the way for leveraging CSF sAXL levels as a biomarker to aid ALS disease stratification.

## Teaser

Spatafora *et al*. provided first evidence of a significant association between increased CSF levels of soluble AXL (sAXL) at diagnosis and disease progression in amyotrophic lateral sclerosis (ALS). Their findings suggest the implication of AXL pathway in ALS and support CSF sAXL levels as a biomarker for disease stratification.

## Graphical Abstract

**Graphical Abstract:**
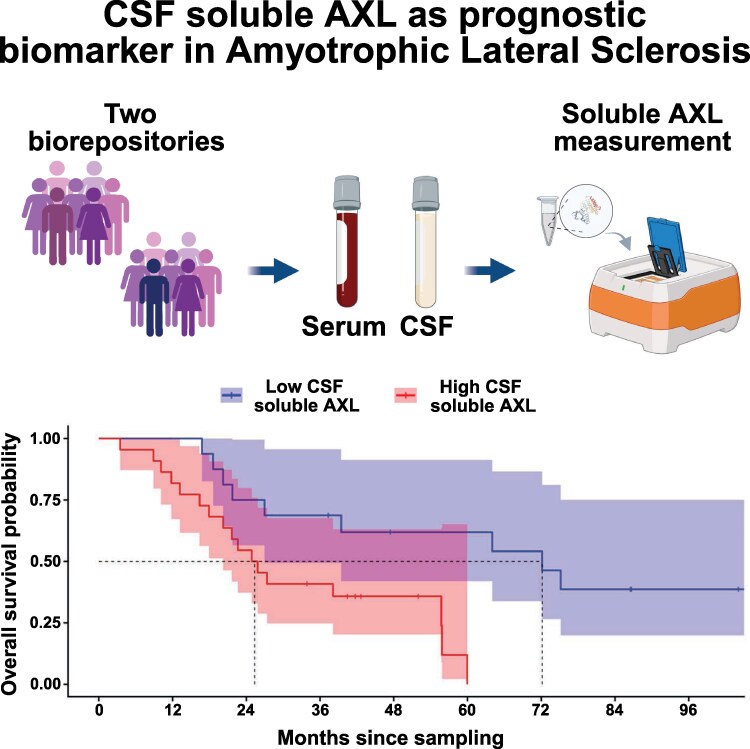

## Introduction

The heterogeneity in clinical phenotype and disease progression in patients with amyotrophic lateral sclerosis (ALS) complicates optimal clinical trial design. Efforts to identify reliable protein biomarkers for patient stratification based on disease prognosis or underlying pathophysiological mechanism have increasingly focused on glial-mediated neuroinflammation, a key contributor to ALS disease progression. Biomarkers reflecting neuroinflammation in ALS include chitotriosidase (CHIT1), chitinase-3-like protein (YKL-40) and glial fibrillary acidic protein.^1,2^ AXL, a receptor tyrosine kinase with neuroprotective and immunomodulatory functions,^3-5^ recently attracted great interest as a novel biomarker in Alzheimer’s disease (AD). Indeed, AXL signalling in reactive microglia mediates the recognizing and engulfing of beta-amyloid plaques in AD.^6^ Upregulation of *Axl* mRNA has been reported also in reactive microglia of the brain and spinal cord in a mouse model of TDP-43 proteinopathy, a pathological hallmark of ALS.^7^ Additionally, *AXL* mRNA is expressed in neurons,^8^ including motor neurons,^9^ and highly enriched in the meninges, pericytes and astrocytes within the human spinal cord,^9^ suggesting a role not only in neuronal survival but also in regulating the blood brain barrier and glial reactivity in neuronal districts affected by ALS. Modulation of AXL signalling can occur through the cleavage of its extracellular domain by sheddases (like TACE, i.e. TNF-alpha converting enzyme), which are upregulated under proinflammatory conditions like in ALS.^10^ This cleavage results in the release of a soluble ectodomain (sAXL) and prevents the membrane-bound portion of AXL from transducing intracellular signals. A recent study in AD patients showed that serum levels of sAXL, in contrast to YKL-40, inversely correlated with cognitive performance and structural integrity in medial temporal brain regions.^11^ Moreover, CSF sAXL levels were significantly increased in AD patients and positively correlated with markers of neurodegeneration (i.e. neurofilament light chain and tau), as well as with cognitive derangement.^12^ Given the involvement of sheddases in neuroinflammation in ALS,^10^ and considering the added value of sAXL over YKL-40 observed in AD, we focused our investigation on whether sAXL (in CSF or blood) may serve as a valuable biomarker also in ALS.

## Materials and methods

### Biosamples selection

Serum and CSF samples, collected according to standard operating procedures and stored in the biorepositories of the KU Leuven Neurobiobank and the Fondazione IRCCS Istituto Neurologico ‘Carlo Besta’ (FINCB), were used in this study. All samples are clinically validated. Further details on the KU Leuven and the FINCB biobanks are provided in the supplementary methods.

### Biomarker measurement in CSF and blood samples

sAXL levels were measured using the Simple Plex Human Axl Cartridge (cat. nr. SPCKB-PS-000472) on the Ella™ automated immunoassay system (ProteinSimple). Each sample was thawed on ice and then centrifuged for 5 min at 12 000 rpm at 4°C. The supernatant was diluted 1:10 (serum) or 1:20 (CSF) in Sample Diluent buffer. Then, 48 μl of each diluted sample was loaded in one well of the immunoreaction cartridge. During the reaction, the machine splits each sample into three microfluidic glass nanoreactors (GNRs) coated with the capture antibody, providing analyte measurements in triplicate for each sample added to the cartridge. Results were reported as averages of the concentrations calculated based on the RFU (relative fluorescence units) measured in each sample GNR triplicate. GNR filtering was applied, with a CV threshold set at 5%. Based on the software readout, for GNR population %CV exceeding the threshold, the outlier GNR value was removed based on the minimum population standard deviation of the three possible GNR pair combinations. Quality controls were included in each analysis to verify inter-assay variability, resulting in a CV of 18.67% for serum and 10.12% for CSF. Levels of pNfH, NfL and YKL-40 had been previously quantified through ELISA with a commercial kit from EUROIMMUN (cat. nr. EQ 6561-9601), from Uman Diagnostics (cat. nr. 10-7001), and Quidel (cat. nr. 8020), respectively, as previously described.^13^ Therefore, the measurements of pNfH, NfL, and YKL-40 were retrieved from the KU Leuven Neurobiobank database and used for the statistical analyses.

### Statistics

Statistical analysis was performed via R software (v4.4.1, The R foundation for Statistical Computing), unless stated otherwise. Shapiro–Wilk test and D’Agostino–Pearson test were used for assessing the normality of data. Difference between groups (demographic data) was identified using unpaired *t*-test or Wilcoxon rank-sum test in the case of comparisons between two groups, or by using analysis of variance (ANOVA) followed by Dunnet’s post-hoc test or Kruskal–Wallis test followed by Dunn’s post-hoc test in case of comparison between more than two groups.

For analyses involving serum sAXL levels, mixed-effect analysis was performed first via Lumivero XLSTAT software [version 2024.1.1 (1419)], setting the disease status (ALS or healthy controls, HC) as a fixed effect and the two biorepositories (namely KU Leuven and the FINCB) as the random effects. For analyses involving CSF sAXL levels, adjustments were made for age and gender using multiple linear regression models (*stats* R package). Similarly, adjustments for age were made for analyses involving serum sAXL levels. These models were used for evaluating the difference in CSF or serum sAXL between ALS patients and HC, as well as between all included patients in the two cohorts, and to assess the association between CSF sAXL and clinical parameters in ALS patients. Transformations, i.e. natural log (ln) or square root, were used if required to meet model assumptions (i.e. normality and homoscedasticity of residuals). CSF sAXL and age in the ALS cohort were standardized through conversion to *z*-scores (based on the mean and standard deviations of biomarker values) in the models used to analyse the association of the biomarker with clinical parameters. Figures showing the difference in serum or CSF sAXL levels between different disease groups were constructed using Graphpad Prism software (version 10.3.1(509)). Receiver operating characteristic (ROC) analysis was used to determine the discriminative potential of sAXL in CSF (adjusted for age and gender) and serum (adjusted for age) between ALS patients and HC using logistic regression models (*stats* and *pROC* R package). Youden index analysis was used to determine the optimal threshold in CSF to distinguish ALS patients from HC (unadjusted analysis). Survival (*survival* R package) was estimated using Kaplan–Meier curve; the log-rank test was conducted to determine differences between survival curves after stratification based on Youden index threshold for CSF sAXL. A Cox proportional-hazards regression survival model was calculated using standardized CSF sAXL levels and DPR as a categorical variable (unadjusted model and adjusted for age and gender). The proportional hazards assumption was met for Cox regression models. Covariate-adjusted partial Spearman rank correlation coefficients were calculated to estimate the association between CSF sAXL and serum sAXL (*n* = 115), CSF NfL (*n* = 82), CSF pNfH (*n* = 75), and CSF YKL-40 (*n* = 63) in the Leuven Neurobiobank CSF cohort (*ppcor* R package).

See supplementary methods for equations of linear regression models for covariate-adjustments.

### Study approval

The study was approved by the institutional review board at San Matteo Hospital, University of Pavia (Protocol ID: 2022-3.11/788 approved on Jan 10th 2023) and at KU Leuven (Protocol ID: S67768 approved on May 8th 2023). Study procedures were in accordance with institutional guidelines.

## Results

We measured sAXL levels in fluid biosamples obtained from two biorepositories: the KU Leuven Biorepository (CSF and serum) and the Fondazione IRCCS Istituto Neurologico ‘Carlo Besta’, hereafter called FINCB (serum), including 107 ALS patients (107 serum and 38 CSF samples), 76 HC (76 serum and 23 CSF samples), 25 AD patients (25 serum and CSF samples), 22 patients with multiple sclerosis (MS; 22 serum and 12 CSF samples), and 51 patients with ALS disease mimicking disorders (ALSdm, 45 serum and 24 CSF samples). The demographics of the population are provided in Table 1. Each gender was equally represented in all experimental groups except for ALSdm. In the CSF and serum sub cohorts, the ALS and AD populations were significantly older than HC whereas MS population was younger than HC in the serum sub cohort. Therefore, for ALS, AD and MS sub cohorts, the HC group was not age matched. A positive association between CSF sAXL levels and age at lumbar puncture (Spearman’s *ρ* = 0.329, *P* = 0.0002), and between serum sAXL levels and age at blood sampling (Spearman’s *ρ* = 0.155, *P* = 0.0098) was observed (Supplementary Fig. 1A, B, respectively). Moreover, in the whole CSF sub cohort, sAXL levels were higher in males compared with females (Supplementary Fig. 1C; Wilcoxon rank sum test, *P* = 0.005), whereas no difference between genders was observed for sAXL levels in serum (Supplementary Fig. 1C). Therefore, we adjusted all CSF sAXL analyses for age and sex, whereas serum sAXL analyses were adjusted only for age, using multiple linear regression models.

**Table 1:** Demographic information of the cohorts analysed

		Serum					CSF				
		HC	ALS	AD	MS	ALSdm	HC	ALS	AD	MS	ALSdm
*N*		76	107	25	22	45	23	38	25	12	24
Gender (M/F)		36/40	52/55	12/13	11/11	23/22	12/11	20/18	12/13	6/6	16/8
		*Chi-square test for gender: P = 0.9962*					*Chi-square test for gender: P = 0.7339*				
Age (y)	Median	**51**	**62^d^**	**68^d^**	**37^c^**	**56**	**49**	**62^e^**	**68^f^**	**37.5**	**55**
	Range	(20–80)	(35–88)	(51–82)	(21–63)	(19–86)	(26–71)	(36–82)	(51–82)	(21–58)	(19–78)
	M	**50**	**62^c^**	**68.5^c^**	**35^a^**	**54**	**49.5**	**62^a^**	**68.5^b^**	**35**	**53.5**
		(20–73)	(36–88)	(51–82)	(21–58)	(19–80)	(26–71)	(36–72)	(51–82)	(21–58)	(19–78)
	F	**52.5**	**61^b^**	**67 ^b^**	**38^b^**	**59.5**	**46**	**62.5^b^**	**67^c^**	**38**	**57**
		(26–80)	(35–82)	(51–80)	(26–63)	(24–86)	(26–68)	(42–82)	(51–80)	(35–55)	(24–74)

Median (highlighted in bold) and range are given. ^a^*P* < 0.05, ^b^*P* < 0.01, ^c^*P* < 0.001, and ^d^*P* < 0.0001; ordinary one-way ANOVA followed by Dunnet’s post-hoc test versus HC. ^e^*P* < 0.01, ^f^*P* < 0.0001; Kruskal-Wallis followed by Dunn’s post-hoc test versus HC.

**Figure 1:**
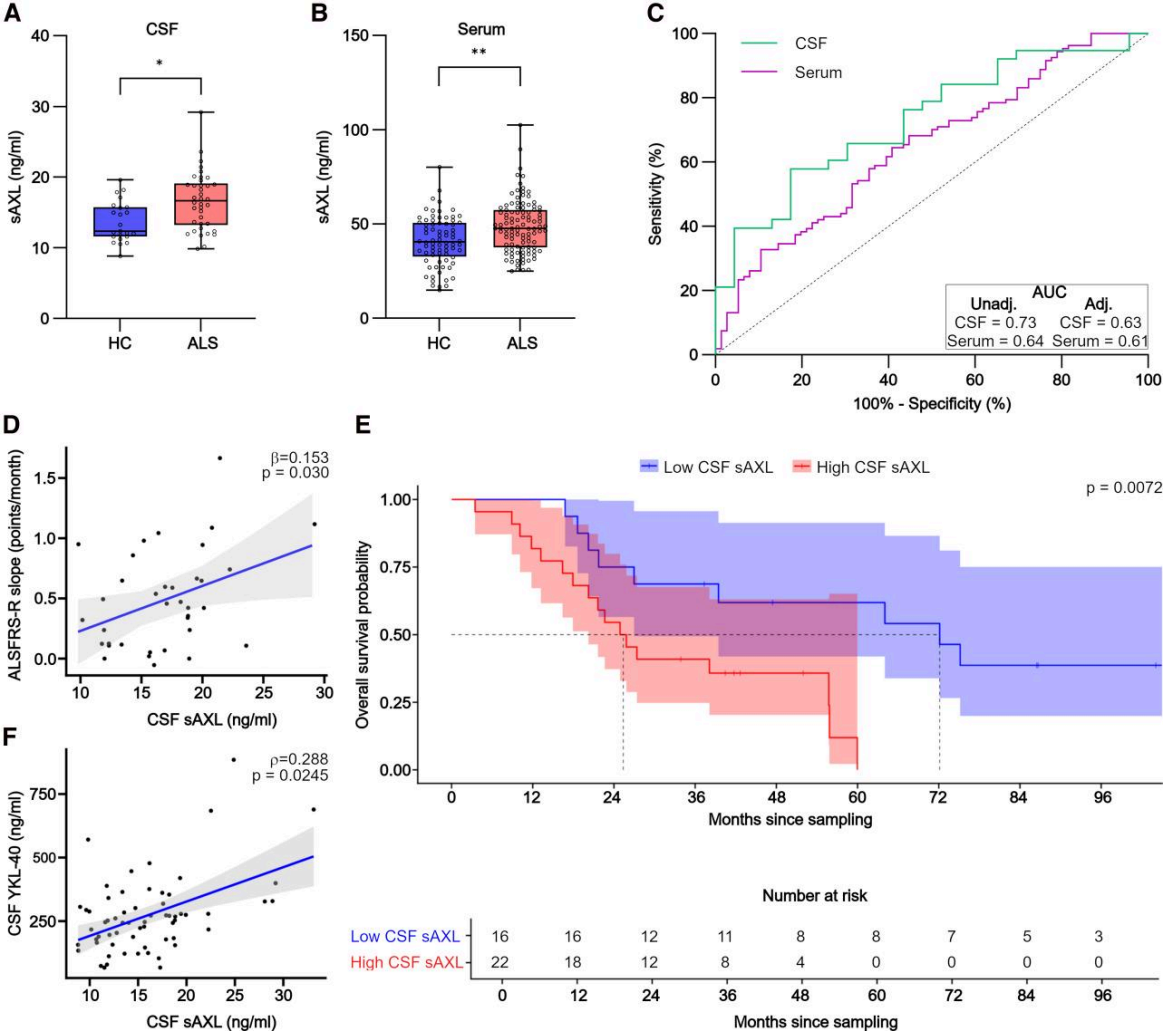
sAXL levels are significantly increased in the CSF and serum of ALS patients at diagnosis. (**A**) Boxplot showing sAXL in CSF of ALS patients (*n* = 38) compared with healthy controls (HC, *n* = 22). *P < 0.05; age- and sex-adjusted regression *t*-test. (**B**) Boxplot showing sAXL in serum of ALS (*n* = 107) compared with HC (*n* = 76). ** *P* < 0.01; age-adjusted regression *t*-test. Boxplots in **A** and **B** highlight the 25th percentile, median value, and 75th percentile. The whiskers extend to the most extreme data points within 1.5 times the interquartile range from the quartiles, while outliers beyond this range, if any, are shown. Each dot in the graphs is a subject. (**C**) Unadjusted receiver operating characteristic (ROC) curve indicating the diagnostic performance of sAXL measured in the CSF or serum in ALS patients. The AUC of the unadjusted and adjusted ROC are reported on the graph. (**D**) Scatter plot showing the relationship between CSF sAXL with the slope of the revised ALS Functional Rating Scale (ALSFRS-R) score over time (months) in ALS patients (*n* = 36). Age- and sex-adjusted regression coefficient (*β*) with corresponding *P*-value is shown on the graph. The ALSFRS-R slope underwent logarithmic transformation on the *y*-axis (see Methods section). Each dot in the graph represents one patient. (**E**) Survival analysis from time of lumbar puncture (LP) expressed in months, with ALS patients stratified based on the optimal CSF sAXL threshold of 16.034 ng/ml, resulted in a significant difference (log-rank test, *P* = 0.007) in survival probability between the Low CSF sAXL (<16.034 ng/ml; median survival 72.1 months, *n* = 16) and High CSF sAXL (≥16.034 ng/ml; median survival 25.4 months, *n* = 22) group. (**F**) Scatter plot showing the relationship between CSF levels of sAXL and YKL-40 in tot. *n* = 63 subjects, including patients affected by ALS (*n* = 18), patients with non-ALS pathologies (*n* = 33) and HC (*n* = 12). Age- and sex-adjusted partial Spearman’s *ρ* with corresponding *P*-value is shown on the graph. Each dot in the graph represents one patient.

As shown in Fig. 1A, we measured a significant increase of CSF sAXL in ALS patients compared with HC (*β* = 0.148, *P* = 0.039; average CSF sAXL levels in ALS = 16.65 ng/ml versus HC = 13.64 ng/ml); no differences in sAXL levels were observed between males and females within the ALS sub-cohort (Table 2). We additionally compared CSF sAXL levels between HC and patients with other neurological diseases and confirmed a statistically significant increase only in AD patients (*β* = 0.202, *P* = 0.022) in line with literature data^12^ (Supplementary Fig. 1D). Furthermore, we observed a positive association between sAXL levels in matched CSF and serum samples (covariate-adjusted partial Spearman’s *ρ* = 0.211, *P* = 0.024; Supplementary Fig. 1G).

**Table 2:** Comparison of sAXL levels between males and females

		Serum					CSF				
		HC	ALS	AD	MS	ALSdm	HC	ALS	AD	MS	ALSdm
*n* (M/F)		36/40	52/55	12/13	11/11	23/22	12/10	20/18	12/13	6/6	16/8
sAXL (ng/ml)	M	**42.26**	**50.01**	**47.05**	**43.75**	**40.22**	**13.54**	**17.54**	**20.99**	**16.29**	**17.47**
		(14.89–80.16)	(25.64–102.6)	(28.90–49.49)	(36.12–51.91)	(20.43–69–76)	(10.51–19.61)	(11.76–29.20)	(10.39–28.09)	(12.81–24.88)	(11.15–33.14)
	F	**39.00**	**47.42**	**45.03**	**33.49**	**41–27**	**12.06**	**16.22**	**16.53**	**12.13**	**11.45**
		(19.53–63–50)	(29.94–75.99)	(33.28–74.43)	(27.94–46.32)	(22.11–58–52)	(8.81–18.17)	(9.83–20.12)	(9.96–24.13)	(9.00–21.12)	(8.79–28.88)
*P*-value		0.595	0.524	0.574	0.0002	0.509	0.404	0.137	0.181	0.065	0.106
Test		*t* Test	Mann–Whitney	Mann–Whitney	Mann–Whitney	*t* Test	*t* Test	*t* Test	*t* Test	Mann–Whitney	Mann–Whitney

For each sub cohort, median (highlighted in bold) and range are given. The comparison was performed between sAXL levels in males versus females within each sub cohort.

For analysing serum sAXL levels, a mixed-model analysis confirmed that the biorepository site of sampling did not confound sAXL assessment between HC and ALS subjects (mixed-model analysis, CI(%) = 95; Analysis Centre: KU Leuven, *P* = 0.333; Besta Institute, *P* = 0.333). A significant increase of sAXL levels was observed in the serum of ALS patients compared with HC (*β* = 0.517, *P* = 0.002, average serum sAXL levels in ALS = 48.72 ng/ml versus HC = 40.89 ng/ml, Fig. 1B). No differences were observed in serum sAXL levels across non-ALS pathologies sub cohorts, compared with the HC group (Supplementary Fig. 1E). No gender-related differences in sAXL levels were observed in serum across all sub-cohorts, except for MS patients, in whom males showed higher sAXL levels compared with females (Table 2).

We performed ROC analysis to investigate the potential of sAXL in both CSF and serum to distinguish ALS (Fig. 1C) or AD patients (Supplementary Fig. 1F) from HC. The area under the adjusted ROC curve (AUC) was 0.63 (95% CI 0.48–0.77) for CSF sAXL and 0.61 (95% CI 0.53–0.68) for serum sAXL in ALS, whereas in AD it was 0.66 (95% CI 0.51–0.81) and 0.52 (95% CI 0.40–0.64) for sAXL in CSF and serum, respectively.

Interestingly, we observed a positive association between CSF sAXL levels at diagnosis and longitudinal disease progression rate (DPR) in ALS patients, estimated by the slope of the ALS Functional Rating Scale (ALSFRS-R) score over time, adjusted for age and gender (*β* = 0.153, *P* = 0.030, Fig. 1D), while no significant association was found with ALSFRS-R score (*P* = 0.202) at diagnosis and baseline DPR (*P* = 0.241), or with disease duration (*P* = 0.525). Therefore, we decided to stratify ALS patients based on CSF sAXL levels, using 16.034 ng/ml as threshold (calculated by applying Youden index analysis of the unadjusted ROC curve). Interestingly, Kaplan–Meier survival analysis revealed that ALS patients with low (<16.034 ng/ml) CSF sAXL levels (*n* = 16) have a significantly longer survival (median survival of 72.1 months, log-rank test, *P* = 0.007) compared with ALS patients with high (>16.034 ng/ml) CSF sAXL levels (*n* = 22, median survival of 25.4 months) (Fig. 1E). Notably, demographics and the clinical parameters—ALSFRS-R and DPR—between these two ALS subgroups were not different (Table 3). Given that DPR is currently considered an established prognostic biomarker for predicting survival in ALS,^14-16^ we next evaluated whether CSF sAXL levels provide additional or stronger predictive value relative to a clinical measure. In an age- and gender-adjusted Cox regression model (*n* = 32; 24 events), higher CSF sAXL levels were significantly associated with shorter survival (HR 2.02, 95% CI 1.22–3.31; *P* = 0.006), whereas baseline DPR was not (HR 1.35, 95% CI 0.87–2.08; *P* = 0.178) (Table 4).

**Table 3:** Patient demographics of the CSF ALS cohort, stratified in low CSF sAXL and high sAXL groups according to the optimal threshold (16.034 ng/ml)

	Low sAXL	High sAXL	*P*-value
*N* (%)	16 (42.1%)	22 (57.9%)	
Age (years)	56.5	63.4	0.086^a^
	(36.5–78.9)	(50.1–82.1)	
Gender (male/female)	8/8	12/10	1^b^
Bulbar onset *N* (%)	4 (25%)	6 (27%)	1^b^
ALSFRS-R at lumbar puncture (points)	38	37	0.478^a^
	(29–45)	(27–46)	
Disease duration (months)	14.3	12.8	0.715^c^
	(3.5–66.1)	(3.6–54.9)	
Baseline DPR (points/month)	0.655	0.648	0.495^c^
	(0.113–2.000)	(0.217–3.200)	
ALSFRS-R slope (points/month)	−0.200	−0.657	0.153^c^
	(−1.660–0.000)	(−4.290–0.055)	
Censoring (event/censor)	9/7	17/5	0.306^b^

Median value and range are given. *P*-values correspond to unpaired *t*-test^a^, Chi-square test^b^, and Wilcoxon rank-sum test^c^. *P*-value < 0.05 was considered significant.

**Table 4:** Univariate and multivariate cox proportional-hazards regression for CSF sAXL and DPR in ALS cohort

	Univariate unadjusted			Univariate adjusted				
	Cox regression			Cox regression				
Variable	HR (95% CI)	*z*-statistic	*P-*value^a^	HR (95% CI)	*z*-statistic	*P-*value^a^	AIC	*P-*value^b^
CSF sAXL	1.83 (1.15–2.89)	2.543	0.010	2.02 (1.22–3.31)	2.742	0.006	–	–
*Age*	–	–	–	0.80 (0.53–1.23)	−1.008	0.314		
*Gender (M)*	–	–	–	1.82 (0.44–2.20)	−0.048	0.961		
DPR	1.28 (0.85–1.91)	1.174	0.240	1.35 (0.87–2.08)	1.346	0.178	138.94	–
*Age*	–	–	–	0.95 (0.59–1.52)	−0.235	0.814		
*Gender (M)*	–	–	–	1.82 (0.76–4.34)	0.343	0.179		
CSF sAXL	1.87 (1.18– 2.94)	2.686	0.007	1.97 (1.21–3.20)	2.710	0.007	133.74	0.007
*DPR*	1.36 (0.93–1.98)	1.607	0.108	1.30 (0.86–1.95)	1.252	0.211		
*Age*	–	–	–	0.73 (0.43–1.22)	−1.196	0.232		
*Gender (M)*	–	–	–	1.30 (0.53–3.19)	0.571	0.568		

Data are represented as the HR (95% CI) in the full ALS cohort (*n* = 38) for sAXL, and in the subset of ALS patients with ALSFRS-R data within 3 months of lumbar puncture (*n* = 32) for DPR and multivariate model. Unadjusted and adjusted model parameters are given. *P*-values correspond to the Wald test for HR^a^ and log-likelihood ratio for model fit^b^. The log-likelihood ratio test was performed by comparing the log-likelihood of the multivariate model against the DPR-only model. In the table, Akaike Information Criterion (AIC) and *P*-value are shown. *P*-value < 0.05 was considered significant.

Adding sAXL to a model already containing DPR significantly improved model fit (ΔLR *χ*^2^ = 7.20; *P* = 0.007) and reduced AIC (from 138.94 to 133.15), indicating that CSF sAXL levels represent a strong and independent predictor of survival in ALS, providing complementary information to clinical measures.

Furthermore, we highlighted a positive association between CSF sAXL and the glial marker YKL-40 (covariate adjusted partial Spearman’s *ρ* = 0.288, *P* = 0.024, Fig. 1F). CSF sAXL was also positively associated with CSF NfL (covariate-adjusted partial Spearman’s *ρ* = 0.286, *P* = 0.010) but not with phosphorylated neurofilament heavy chain (pNfH, covariate-adjusted partial Spearman’s *ρ* = 0.224, *P* = 0.056) (Supplementary Fig. 1H, I, respectively), which are well-established markers of neurodegeneration.

## Discussion

This study provides the first evidence of sAXL alterations in CSF and serum of ALS patients at diagnosis, implicating the AXL signalling pathway in ALS pathology. Noteworthy, we also observed increased CSF sAXL levels in AD, consistent with previous reports.^12^

Despite the limited sample size of our CSF cohort, higher CSF sAXL levels were significantly associated with a more rapid clinical decline, as measured by ALSFRS-R slope, and shorter survival. Importantly, the prognostic value of sAXL remained significant when accounting for an established clinical predictor, such as baseline DPR, underscoring the potential utility of sAXL as a novel independent prognostic biomarker in ALS. These findings warrant further validation in large, multicentre longitudinal cohorts. Interestingly, these results diverge from findings in AD, where increased CSF sAXL levels have been associated with higher cognitive reserve.^12,17^ This discrepancy may reflect a different role played by neuroinflammatory responses in the two pathologies or, alternatively, sAXL changes might reflect the involvement of AXL pathway in different neuropathological events in ALS versus AD. Indeed, it has been hypothesized that in AD the increase of CSF sAXL, alongside sTYRO3 and sTREM2, might reflect the engagement of disease-associated microglia (DAM), responsible for efficient engulfment and detoxification of beta-amyloid plaques and/or dying neurons.^12,17^ In contrast, the association between elevated CSF sAXL and worse prognosis in ALS may reflect maladaptive neuroinflammatory responses or neurodegeneration.

In human spinal cord, AXL is expressed not only in microglia, but also in meninges, pericytes, astrocytes, and motor neurons.^9^ Thus, increased CSF sAXL levels may arise from various sources, potentially driven by higher sheddase activity, which has been documented in ALS mouse models.^10^ Since AXL signalling is important not only for myelination and immune function but also for neuronal survival, it can be hypothesized that increased sAXL levels in ALS might reflect not only glial reactivity but also neuronal demise. This hypothesis warrants further investigation, leveraging ALS animal models to better correlate the changes in sAXL levels with specific neuropathological events occurring during disease progression.

Increased serum sAXL levels have been observed in MS patients displaying milder symptoms at onset,^18^ suggesting a correlation between sAXL and delayed demyelination. Although we highlighted increased sAXL levels in serum of ALS patients, we observed a modest association between sAXL measured in matched CSF and serum samples from the same subject. Outside the CNS, AXL is primarily expressed in myeloid cells and tissue-resident macrophages,^19^ and it has been recently detected also on extracellular vesicles circulating in peripheral blood.^20^ Therefore, further studies are needed to determine whether sAXL levels in CSF and serum in ALS reflect distinct cellular sources associated with different pathological events occurring in the CNS versus peripheral tissues. Moreover, recent studies on the plasma clearance of sAXL highlighted the need to additionally account for perturbations in liver and kidney functions when evaluating sAXL as biomarker in peripheral blood.^21^

We additionally found that CSF sAXL levels moderately correlated with YKL-40, a marker of neuroinflammation, and NfL, a marker of neurodegeneration, but not with pNfH. These results suggest that sAXL may reflect overlapping but not identical pathological processes. Whether sAXL directly reflects disease-specific, potentially druggable, pathological changes in glial reactivity in ALS remains to be determined. An important question is whether combining CSF sAXL with other established biomarkers enhances its utility for patients’ stratification, or whether sAXL levels in CSF or serum change over time during disease progression, supporting its utility as disease monitoring tool. Importantly, follow-up studies should also include the measurement of other members of the TAM receptor family (sTYRO3 and sMERTK) as well as the DAM marker sTREM2 to deepen our understanding of the role played by these signalling pathways in ALS, as compared with other neurodegenerative conditions such as AD. Moreover, cohorts with clinical data on the cognitive status of ALS patients are needed to explore potential correlations between blood or CSF sAXL levels and cognitive impairment in ALS, as was observed by other authors in AD.^12^ Indeed, cognitive decline is increasingly recognized as a contributing factor to ALS heterogeneity.^22^ Therefore, sAXL may represent a new valuable biomarker in disease stratification.

In summary, we identify sAXL as novel, promising biomarker for disease progression and prognosis in ALS. By capturing complementary information to existing clinical measures, sAXL holds promise for improving patients’ stratification and advancing our understanding of disease mechanisms involving AXL signalling in ALS.

## Supplementary Material

fcag086_Supplementary_Data

## Data Availability

The authors confirm that the data supporting the findings of this study are available within the article and its Supplementary material.
