# Supplementary material for: Increased CSF levels of soluble AXL at diagnosis correlate with poor prognosis in patients affected by amyotrophic lateral sclerosis
Source: Brain Commun. 2026 Mar 30;8(2):fcag086. doi: 10.1093/braincomms/fcag086 (PMC13034920; doi:10.1093/braincomms/fcag086)
Supplement: fcag086_Supplementary_Data [file fcag086_supplementary_data.zip › Supplementary_material.docx]

**Supplemental Material**

**Increased CSF levels of soluble AXL at diagnosis correlate with poor prognosis in patients affected by amyotrophic lateral sclerosis**

Mauro G. Spatafora**^†^**, Jonas Dubin**^†^**, Teuta Domi, Raffaella Lombardi, Paolo Cabras, Eleonora Dalla Bella, Monica Consonni, Angelo Quattrini, Manuela Verri, Giuseppe Lauria, Philip Van Damme**^†^**, Koen Poesen**^†^**, Nilo Riva**^†^**, Marco Peviani**^†^**

**^†^** Mauro G. Spatafora, Jonas Dubin, Philip Van Damme, Koen Poesen, Nilo Riva and Marco Peviani contributed equally to this work.

Contents:

- Supplementary methods
- Supplementary Figure 1
- Author Contributions

**Supplementary methods**

Biorepositories.

*Leuven Neurobiobank.*

Serum and CSF were sampled from patients referred to the University Hospitals Leuven between 2014 and 2022 and stored in the Leuven Neurobiobank. Patients with ALS were diagnosed according to the revised El Escorial and Awaji criteria by an expert neurologist (PVD). A diagnosis of ALS-FTD was reported for n = 2 subjects within the ALS cohort. Additionally, mutations in ALS-associated genes were reported for n = 11 patients (*C9ORF72*, n = 6; *SOD1*, n = 4; *TARDBP*, n = 1). The disease duration at sampling was defined as the period between sampling and time of symptom onset reported by the patient. The diagnostic delay was defined as the time between symptom onset reported by the patient and the diagnosis. The baseline disease progression rate was defined as the difference between the maximum ALS Functional Rating Scale (ALSFRS-R) score and the ALSFRS-R score closest to the date of CSF sampling, divided by the disease duration at the date of ALSFRS-R. A second ALSFRS-R score was obtained during follow-up. The slope of the ALSFRS-R score over time was calculated as the difference between the last ALSFRS-R score in follow-up and the ALSFRS-R score closest to sampling, divided by the time difference between the two ALSFRS-R scores. The median time between LP and ALSFRS-R score closest to sampling was 0 months (range: -7.1 – 9.6 months, n = 36). Survival was defined as the timespan between sampling and date of death. The date of survival analysis (May 12th 2024) was used for censoring when the patient was still alive. Moreover, additional cohorts of patients were included whose serum and/or CSF were sampled and who received a diagnosis of Alzheimer’s Disease (AD), multiple sclerosis (MS), or were categorized as ALS mimicking disorder (ALSdm), i.e. patients that displayed symptoms resembling ALS, a diagnosis that was then excluded after a thorough clinical examination. Patients with headaches and in follow-up no evidence of neurodegeneration or neuroinflammation were used as healthy controls (HC).

*Fondazione IRCCS Istituto Neurologico “Carlo Besta” (FINCB) biobank.*

At the collection time, demographics and clinical history information of all ALS patients were registered and a neurological evaluation was performed by an expert neurologist. Patients were staged according to King's clinical staging system, and classified into eight different ALS phenotypes, in accordance with previously published criteria. For this work, only patients with a classical (spinal onset) or bulbar phenotype were included in the analysis. A neuropsychological assessment, performed as recommended by the Diagnostic Criteria for the Behavioral Variant of frontotemporal dementia (FTD) and the ALSFTD Consensus Criteria, was performed for all the patients. A diagnosis of ALS-FTD was reported for n = 4 subjects within the ALS cohort. Additionally, mutations in ALS-associated genes were reported for n = 9 patients (*C9ORF72*, n = 6; *SOD1*, n = 1; *FUS*, n = 2). Patients with no evidence of neurodegeneration or neuroinflammation were used as HC.

Equations of linear regression models for covariate-adjustments.

The predictor variable for which regression coefficients were reported is indicated in bold.

*(1)- Difference in CSF sAXL between ALS and HC*

*lm(CSF sAXL) ~* ***ALS*** *+ Age + Gender*

*(2) - Difference in CSF sAXL between AD, MS, ALSdm and HC*

*lm(CSF sAXL) ~* ***AD + MS + ALSdm*** *+ Age + Gender*

*(3) - Difference in serum sAXL between ALS and HC*

*sqrt(Serum sAXL) ~* ***ALS*** *+ Age*

*(4) - Difference in serum sAXL between AD, MS, ALSdm and HC*

*lm(Serum sAXL) ~* ***AD + MS + ALSdm*** *+ Age*

*(5) - Association between CSF sAXL and clinical features in ALS*

*lm(disease duration) ~* ***CSF sAXL (z-scored)*** *+ Age (z-scored) + Gender*

*ALSFRS-R ~* ***CSF sAXL (z-scored)*** *+ Age (z-scored) + Gender*

*lm(baseline disease progression) ~* ***CSF sAXL (z-scored)*** *+ Age (z-scored) + Gender*

*transformed(ALSFRS-R slope)^a^ ~* ***CSF sAXL (z-scored)*** *+ Age (z-scored) + Gender*

^a^The transformation for the ALSFRS-R slope was:

- sign(ALSFRS-R slope) * ln[abs(ALSFRS-R slope)+1].

**Supplementary Figure 1.**

**
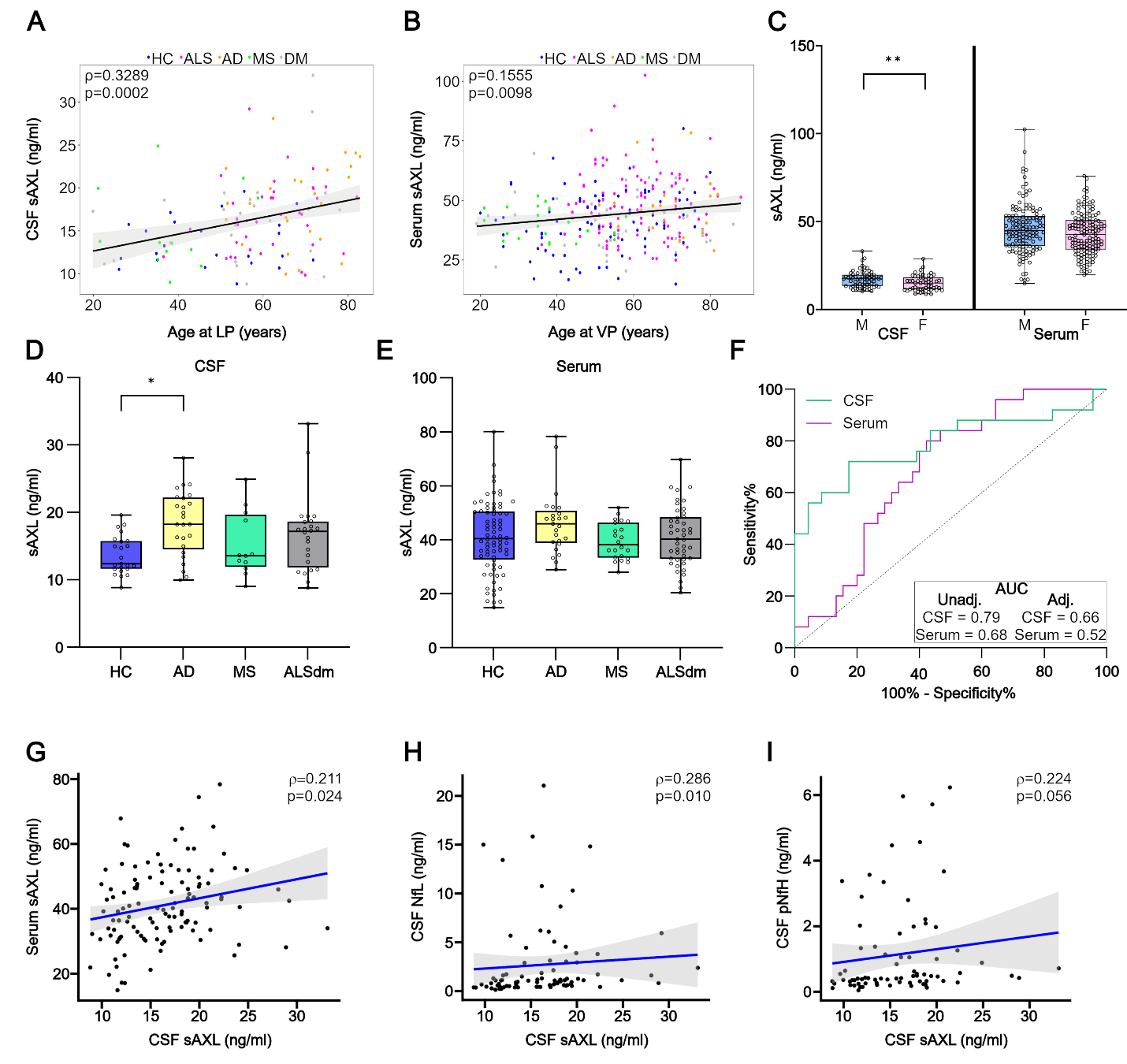
**

**Assessment of sAXL levels in CSF and serum of patients affected by neurodegenerative and neuroinflammatory disorders, and correlation of sAXL with other biomarkers.**

(**A, B**) Scatter plots showing the relationship between CSF (**A**) or serum (**B**) levels of sAXL and age at biosample withdrawal. Spearman’s rank correlation with corresponding p-value is shown on top of each plot. Each dot represents a subject belonging to one of the following sub cohorts, highlighted with different colors in the graphs: Healthy controls (HC, CSF: n = 23, serum: n = 76), subjects affected by Amyotrophic Lateral Sclerosis (ALS, CSF: n = 38, serum: n = 107), Alzheimer’s disease (AD, n = 25), Multiple Sclerosis (MS, CSF: n = 12, serum: n = 22) and ALS mimicking disorders (ALSdm, CSF: n = 24, serum: n = 45). (**C**) Boxplots showing sAXL levels in CSF or serum in female or male subjects from the whole patients’ cohort analyzed. ** = p < 0.01, Wilcoxon rank sum test. (**D, E**) Boxplots showing sAXL levels in CSF (**D**) or in serum (**E**) of healthy controls (HC n = 22 or 76, respectively), subjects affected by Alzheimer’s disease (AD, n = 25), Multiple Sclerosis (MS, n = 12 or 22, respectively) and ALS mimicking disorders (ALSdm, n = 24 or 45, respectively). * = p < 0.05; linear regression model (adjusted for age and sex, in **D**; adjusted only for age, in **E**). Boxplots in D and E highlight the 25th percentile, median value, and 75th percentile. The whiskers extend to the most extreme data points within 1.5 times the interquartile range from the quartiles, while outliers beyond this range, if any, are shown. Each dot in the graphs is a subject. (**F**) Unadjusted Receiver Operating Characteristic (ROC) curve indicating the diagnostic performance of sAXL measured in the CSF or serum in AD patients. The AUC of the unadjusted and adjusted ROC are reported on the graph. (**G**) Scatter plot showing the relationship between CSF sAXL and serum sAXL in matched patient samples (tot. n = 115, including ALS, n =38, AD, n =25, MS, n =12, ALSdm, n =18 and HC, n =22). Covariate-adjusted partial Spearman’s rank correlation (age- and sex-adjusted) with corresponding p-value is shown on top of the plot. (**H, I**) Scatter plots showing the relationship between sAXL CSF levels and NfL (**H)** or pNfH (**I)** in tot. n = 82 and 75 subjects, respectively, including patients with ALS (n = 31 and 30), non-ALS pathologies (n = 38 and 32) and HC (n = 13 and 13, respectively). Covariate-adjusted partial Spearman’s rank correlation (age- and sex-adjusted) with corresponding p-value is shown on top of each plot. Each dot in the graphs shown in G through I represents one patient.

**Author contribution.**

Conceptualization: M.P., K.P., N.R.

Investigation: M.G.S., J.D., P.C., T.D., R.L.

Resources: M.P., K.P., N.R., P.V.D., M.C., E.D., G.L.

Formal analysis: M.P., M.G.S., J.D.

Writing - Original Draft: M.P., M.G.S., J.D., K.P.

Writing - Review & Editing: M.P., M.G.S., J.D., T.D., M.V., K.P., G.L., A.Q.

Funding acquisition: M.P.
